# Palliative care outpatients in a German comprehensive cancer center—identifying indicators for early and late referral

**DOI:** 10.1186/s12904-022-01114-z

**Published:** 2022-12-12

**Authors:** S. Müller, M. Fink, J. Hense, M. R. Salvador Comino, M. Schuler, M. Teufel, M. Tewes

**Affiliations:** 1 Department of Palliative Medicine, West German Cancer Center Essen, University Hospital Essen, 45147 Essen, Germany; 2 Clinic of Psychosomatic Medicine and Psychotherapy, University of Duisburg-Essen, LVR-Klinikum Essen, 45147 Essen, Germany; 3 Department of Medical Oncology, West German Cancer Center Essen, University Hospital Essen, 45147 Essen, Germany; 4 German Cancer Consortium (DKTK), Partner Site University Hospital Essen, 45147 Essen, Germany

**Keywords:** Outpatient, Palliative Care, Advanced Cancer, Timing of Referral, Symptom Burden

## Abstract

**Purpose:**

Despite that early integration of palliative care is recommended in advanced cancer patients, referrals to outpatient specialised palliative care (SPC) frequently occur late. Well-defined referral criteria are still missing. We analysed indicators associated with early (ER) and late referral (LR) to SPC of an high volume outpatient unit of a comprehensive cancer center.

**Methods:**

Characteristics, laboratory parameters and symptom burden of 281 patients at first SPC referral were analysed. Timing of referral was categorized as early, intermediate and late (> 12, 3–12 and < 3 months before death). Ordinal logistic regression analysis was used to identify factors related to referral timing. Kruskal–Wallis test was used to determine symptom severity and laboratory parameter in each referral category.

**Results:**

LRs (50.7%) had worse scores of weakness, loss of appetite, drowsiness, assistance of daily living (all *p* < 0.001) and organisation of care (*p* < 0.01) in contrast to ERs. The mean symptom sum score was significantly higher in LRs than ERs (13.03 vs. 16.08; *p* < 0.01). Parameters indicative of poor prognosis, such as elevated LDH, CRP and neutrophil-to-lymphocyte ratio (NLR) (*p* < 0.01) as well as the presence of ascites (*p* < 0.05), were significantly higher (all *p* < 0.001) in LRs. In univariable analyses, psychological distress (*p* < 0.05) and female gender (*p* < 0.05) were independently associated with an ER.

**Conclusion:**

A symptom sum score and parameters of poor prognosis like NLR or LDH might be useful to integrate into palliative care screening tools.

**Supplementary Information:**

The online version contains supplementary material available at 10.1186/s12904-022-01114-z.

## Introduction

Advanced cancer patients should be offered palliative care treatment in a timely manner in combination with oncological cancer therapy [1–4]. Multiple studies have shown that integration of any modality of palliative care improves various modalities of patients’ environment, such as quality of life, patient experience, patient and family satisfaction, symptom burden and in the form of less aggressive care at the end of life [5–10].

Various conceptual frameworks have been proposed including a recent Delphi study from Hui et al. [11] involving 60 international experts supporting a combination of trigger-based and clinician-based approaches to timely offer palliative care referral, but not all of them are consistently used [12–18]. A large retrospective cohort study with more than 22,500 patients was able to demonstrate that outpatients had high scores in the Edmonton Symptom Assessment System (ESAS) [19, 20] for tiredness, lack of appetite and impaired wellbeing [21]. Another study also mentioned symptoms like lack of appetite, drowsiness, dyspnoea and fatigue as significant factors that tend to intensify in outpatients at the end of life [22]. Additionally, differences in gender and cancer subtypes were observed regarding specialised palliative care consultations [23]. Furthermore, current studies of different cancer types showed that laboratory data like the neutrophil-to-lymphocyte ratio (NLR) is a predictor for reduced overall survival in cancer patients.

Due to the ongoing process of improving treatment options for advanced cancer patients, cancer is increasingly becoming a kind of "chronic disease" with a prolonged survival time [24]. In this context, outpatient care and treatment options such as specialised palliative care are increasingly coming to the forefront [25]. Therefore, many oncological comprehensive cancer centres (CCC) integrate nowadays a specialised palliative care consultation (SPCC) for outpatients into their routine care [26–28].

One of these CCC in Germany established a SPCC in a high volume outpatient clinic for cancer patients. A median of 60 patients had at least one SPCC every year [29]. During a routine SPCC in germany, the Hospice and Palliative Care Evaluation (HOPE) Symptoms and Problem Checklist is used as common tool for documentation [30].

In a former study, we used a patient reported outcome measurement called MInimal DOcumentation System (MIDOS) in the oncological outpatient setting. We showed that the symptoms “weakness”, “depression” and “anxiety” were predictive factors for patient's request of receiving a SPCC [31].

Although many referral criteria and clinical indicators have been already described, the role of laboratory parameters as predictors of early referral to SPCC remains unexamined. Therefore, our primary objective was to analyse symptom burden using the previously validated HOPE score, clinical indicators and timing of referral to a SPCC in our patient population. Furthermore we explored the role of NLR as a laboratory indicator for early referral to SPCC.

In second point, we hypothesized that the symptom burden would be lower in early referrals. Based on previous studies where the female gender was more common in groups of early referral we hypothesis the gender could be a predictor for early referral [32]. Even gynaecological cancer types were described as factor to be referred earlier to a SPCC [23].

## Methods

### Study design

In this retrospective, non-interventional study based on medical records, we reviewed 310 patients who were referred to the outpatient area of SPCC in a German CCC between November 2013 and December 2020. Documentation at first referral to a SPCC and patients’ characteristics in the medical charts were evaluated. All referring physicians had completed the German hematology and oncology medical specialty. The time of death was obtained from the local residents' registration office.

### Demographics

Participants in our study had to meet the following inclusion criteria: age above 18 years and a histologically confirmed solid cancer type according to the Union for International Cancer Control (UICC) stage IV at the time of first referral to a SPCC.

In addition to demographic information such as sex, age, cancer diagnosis, date of first diagnosis and date of metastasis, we collected information with the HOPE Symptoms and Problem Checklist such as chemotherapy treatment, location of metastasis, the reason for referral to the SPCC listed by the attending physician, the existence of a level of care and the patient's physical condition.

#### Timing of referral

We also documented information related to the timing of referral to palliative care in relation to death as follows: first palliative care referrals more than one year before the patient ‘s death were classified as „early “ (ER); referrals between 3 and 12 months before death were classified as „intermediate “; and referrals made less than 3 months before death were classified as „late “ (LR) [32].

#### HOPE

The German Hospice and Palliative Care Evaluation (HOPE) Symptoms and Problem Checklist is used for standard documentation in German inpatient and outpatient hospice and palliative care services since 1999 [33, 34]. The German Association for Palliative Medicine, the German Association for Cancer and the German Hospice and Palliative Care Association developed and validated this checklist. The HOPE Checklist is utilised by all disciplines involved in the care of patient [31]. This checklist includes 16 different items, eight for physical symptoms (pain, nausea, vomiting, dyspnoea, constipation, weakness, loss of appetite, tiredness), two special nursing problems (wound care, assistance with activities of daily living [ADLs]), four psychological issues (depression, anxiety, confusion, tension), two social topics (organization of care, overburdening of family) and one category for an other problem not previously mentioned. Symptoms are documented in a 4-point-Likert-Scale (0 = none, 1 = mild, 2 = moderate and 3 = strong) by a palliative care nurse [35, 36]. The global sum score for each patient is calculated ranking from minimum 0 to maximum 51 [33]. A low score means no complains, a high score means a high symptom burden.

#### ECOG

The Performance status scale from the Eastern Cooperative Oncology Group (ECOG) is a simple and validated tool commonly used in patients with cancer to quantify general wellbeing, physical status and estimate survival [37, 38]. The graduation is according to a 0 to 5 scale, where 0 indicates optimal health and 5 indicates death. A palliative care nurse documented the ECOG status at the time of first specialised palliative care consultation.

#### Laboratory parameters

We abstracted commonly used laboratory parameters, which includes leucocytes [per nL], neutrophil granulocytes [per nL], neutrophil-to-lymphocyte ratio (NLR), haemoglobin [g/dL], total protein [g/dL], albumin [g/dL], CRP [mg/dL] and LDH [U/L], from the routine database of the hospital information system at the time of initial presentation. We defined laboratory parameters as valid if they were documented within a time interval of 4 weeks from the initial presentation.

### Statistical analyses

Data management and analysis were conducted using the program *Statistical Program for Social Sciences SPSS* version 26.0 (IBM, New York). To characterize the patients at first referral and in context of time of referral we used descriptive statistics. We used univariable logistic regression analyses to find indicators of early and late referral. In addition, multivariate analysis was performed to examine the influence of the cancer type on early referral. One-way ANOVA was used for group-differences. To analyse the HOPE symptom burden (as a sum score and each item separately) and laboratory parameters related to the time of referral Kruskal–Wallis-tests were used. The significance level was set at *p* < 0.05 for all tests.

## Results

From November 2013 to December 2020, a total of 310 patients were referred to an outpatient SPCC. Of these, two patients died before the first presentation and 27 patients did not undergo a specialised palliative care consultation for various reasons. Thus, 281 patients underwent a SPCC. Up to the evaluation period on 20 March 2021, 227 patients had died.

The median age of all patients at first presentation to the SPCC was 62 (18–88) years. Gastrointestinal (*N* = 72; 25.6%), lung (*N* = 62; 22.1%), breast cancer (*N* = 49; 17.4%) and sarcomas (*N* = 41; 14.6%) were the most common tumour entities among our cohort. At first referral, 189 patients (67.3%) were receiving chemotherapy at the time. The majority of patients were in the first (*N* = 74; 26.3%) and second (*N* = 73; 26%) palliative chemotherapy line. The median time from first referral to death was 6.38 months (± 9.02). Cumulatively, 95.2% of patients died within two years after the initial presentation. Detailed demographic information is shown in Table 1.

**Table 1:** Baseline characteristics of outpatients referred at first referral

**Patient Characteristics**	*Total,* *N (%)*	*Late referral*^*a*^*,* *n (%)*	*Intermediate referral*^*a*^*,* *n (%)*	*Early referral*^*a*^*,* *n (%)*
**Number of patients**	*281 (100)*			
**Patients died**	*227 (80.1)*	*115 (50.7)*	*82 (36.1)*	*30 (13.2)*
**Gender**	**281**	**115**	**82**	**30**
Male	116 (41.3)	54 (47.0)	35 (42.7)	6 (20.0)
Female	165 (58.7)	61 (53.0)	47 (57.3)	24 (80.0)
**Age at first presentation, in years**				
Mean ± SD	61.54 (12.13)	62.65 (11.95)	60.55 (14.103)	60.33 (10.694)
Median (range)	62 (18–88)	63 (30–87)	62 (18–88)	59 (28–80)
**Site of primary tumor**	**281**	**115**	**82**	**30**
Gastrointestinal tract	72 (25.6)	35 (30.4)	22 (26.8)	4 (13.3)
Lung	62 (22.1)	31 (27.7)	18 (22.0)	6 (20.0)
Breast	49 (17.4)	15 (13.0)	14 (17.1)	12 (40.0)
Sarcoma	41 (14.6)	15 (13.0)	11 (13.4)	3 (10.0)
Genitourinary	16 (5.7)	7 (6.3)	2 (2.4)	0
Head and neck	15 (5.3)	4 (3.6)	6 (7.3)	2 (6.7)
Other gynaecologic	8 (2.8)	3 (2.7)	2 (2.4)	1 (3.3)
Others^b^	18 (6.4)	5 (4.5)	7 (8.5)	2 (6.7)
**Months after first metastasis**				
Mean ± SD	29.16 (37.27)	24.98 (31.285)	25.09 (28.515)	39.76 (55.912)
Median (range)	15.5 (-48–222)	15 (-2–222)	15 (-9–143)	16 (-48–200)
**Chemotherapy at time of first referral**	**281**	**115**	**82**	**30**
Yes	189 (67.3)	75 (65.2)	63 (76.8)	23 (76.7)
No	92 (32.7)	40 (34.8)	19 (23.2)	7 (23.3)
**Chemotherapy line**	**281**	**115**	**82**	**30**
1st	74 (26.3)	19 (16.5)	24 (29.3)	12 (40.0)
2nd	73 (26.0)	28 (24.3)	27 (32.9)	6 (20.0)
3rd	46 (16.4)	32 (27.8)	8 (9.8)	1 (3.3)
> 3rd	45 (16.0)	23 (20.0)	13 (15.9)	6 (20.0)
None	43 (15.3)	13 (11.3)	10 (12.2)	5 (16.7)

^a^Timing of Referral: early, intermediate and late (> 12, 3–12 and < 3 months before death)

^b^Others: includes hematologic, central nervous system, malignant melanoma, cancer unknown primary (CUP)

As shown in Table 2, almost 60 percent of patients did not have a qualified social care planning at time of referral. Before initial presentation, 81.7 percent of patients had already received pain medication.

**Table 2:** Outpatient’s characteristics referred at first referral

**Patient Characteristics**	*Total,* *N (%)*	*Late referral*^*a*^*,* *n (%)*	*Intermediate referral*^*a*^*,* *n (%)*	*Early referral*^*a*^*,* *n (%)*
**Reason for Referral**	**281**	**115**	**82**	**30**
Pain	95 (33.8)	37 (32.2)	27 (33.0)	8 (26.7)
Social care planning (SCP)	58 (20.6)	27 (23.5)	17 (10.7)	6 (20.0)
Dyspnoea	18 (6.4)	9 (7.8)	7 (8.5)	2 (6.7)
Nutritional advice	17 (6.0)	6 (5.2)	6 (7.3)	1 (3.3)
Psychological distress	16 (5.7)	0	5 (6.1)	6 (20.0)
Fatigue	6 (2.1)	5 (4.3)	1 (1.2)	0
Pain and dyspnoea	10 (3.6)	3 (2.6)	5 (6.1)	1 (3.3)
Pain and SCP	9 (3.2)	4 (3.5)	2 (2.4)	2 (6.7)
Pain and fatigue	9 (3.2)	3 (2.6)	3 (3.6)	2 (6.7)
Others^b^	24 (8.5)	7 (6.1)	6 (7.3)	0
More than two reasons	25 (8.9)	14 (12.2)	3 (3.6)	2 (6.7)
**ECOG**^c^	**280**	**115**	**81**	**30**
0	36 (12.9)	8 (7.0)	13 (16.0)	7 (23.3)
1	141 (50.4)	44 (38.3)	47 (58.0)	20 (66.7)
2	64 (22.9)	38 (33.0)	14 (17.3)	3 (10.0)
3	34 (12.1)	21 (18.3)	7 (8.6)	0
4	5 (1.8)	4 (3.5)	0	0
**Qualified social care planning**	**276**	**111**	**81**	**29**
no level of care	161 (58.3)	61 (55.0)	47 (58.0)	19 (65.5)
existing level of care	115 (41.7)	50 (45.0)	34 (42.0)	10 (34.5)
**Pain premedication**	**279**	**115**	**82**	**29**
NSAID	193 (69.2)	80 (69.6)	60 (73.2)	16 (53.3)
Opioid analgesic WHO II	32 (11.5)	13 (11.3)	7 (8.5)	4 (13.3)
Opioid analgesic WHO III	128 (45.9)	60 (52.2)	35 (42.7)	11 (36.7)
Use of coanalgesics^d^	115 (41.2)	44 (42.6)	31 (37.8)	13 (44.8)
No medication	51 (18.3)	14 (12.2)	17 (20.4)	10 (33.3)

^a^Timing of Referral: early, intermediate and late (> 12, 3–12 and < 3 months before death)

^b^Others: includes physiotherapy, fatigue, edema treatment, itching, incontinence, wound management

^c^ECOG: Eastern Cooperative Oncology Group

^d^Includes antidepressants, antiepileptics and benzodiazepines

Most patients were referred late (*n* = 115; 50.7%), 82 (36.1%) patients were referred intermediate and 30 (13.2%) patients were referred early. Patients who were referred early to palliative care were more likely to have breast cancer (40.0% referred early vs. 3.3–20.0% for other cancer sites) and the reason of referral was mostly related to social care planning (*n* = 6; 20.0%) and psychological distress (*n* = 6; 20.0%). Patients with a late referral presented worse physical condition in terms of ECOG performance status (H = 30.054, *p* < 0.001, *n* = 226).

The single factor variance analysis showed that most of patients who were referred early to a SPCC were female (F = 3.638; *p* < 0.05; *N* = 226). In addition, patients who were referred early were on average younger than in the group of late referrals (60.33 years vs. 62.65 years). Using multivariate analysis, the cancer type (diagnosis) did not predict an early referral (F (1,7) = 1,332; *p* = 0.244). Univariable logistic regression analysis (see Table 3) shows that early referral was associated with pulmonary (95% CI, -1.394 to -0.121; *p* < 0.05), hepatic (95% CI, -1.307 to -0.026; *p* < 0.05) or other visceral metastasis (95% CI, -1.6 to -0.1; *p* < 0.05). Psychological distress (95% CI, -3.156 to -0.226; *p* < 0.05) was another indicator for an early referral. In contrast, late referrals were associated with suffering from ascites (95% CI, 0.126 to 1.839; *p* < 0.05), with a higher NLR (95% CI, 0.038 to 0.173; *p* < 0.01) and a higher ECOG performance status (95% CI, 0.409 to 1.2; *p* < 0.001).

**Table 3:** Univariable analyzes of factors associated with early and late palliative care referral

*Subjects*	*Univariable Analyses*		
	***[95% CI]***	***Wald***	***p*-value**
**Gender**	0.554 [0.041; 1.067]	3.638	0.034
**NLR**^a^	0.105 [0.038; 0.173]	9.295	0.002
**ECOG**^b^	0.804 [0.409; 1.2]	15.872	< 0.001
**Ascites**	0.982 [0.126; 1.839]	5.057	0.025
**Type of metastasis**			
Hepatic	-0.666 [-1.307; -0.026]	4.154	0.042
Pulmonary	-0.758 [-1.394; -0.121]	5.446	0.020
Other visceral^c^	-0.850 [-1.6; -0.1]	4.936	0.026
Cerebral	0.332 [-0.463; 1.127]	0.671	0.413
Bone	0.399 [-0.232; 1.03]	1.537	0.215
**Reason of referral**^d^			
Pain	0.445 [-0.396; 1.285]	1.074	0.300
Social care planning	0.458 [-0.409; 1.326]	1.072	0.301
Dyspnoea	0.649 [-0.52; 1.818]	1.183	0.277
Psychological Distress	-1.691 [-3.156; -0.226]	5.118	0.024
Nutritional advice	0.159 [-1.171; 1.489]	0.055	0.815
Others	-	-	-

^a^NLR: neutrophil-to-lymphocyte ratio

^b^ECOG: Eastern Cooperative Oncology Group

^c^All locations except hepatic metastasis

^d^Only categories with more than 10 patients were considered. Reason of referral was given by the referring attending physician

### Analyses to timing of referral

Symptom burden differed in subgroups of early, intermediate and late referral as shown in Table 4. Patients who were referred late had higher HOPE global sum scores (16.1 ± 6.4 vs. 13.0 ± 5.4; *p* < 0.01) as well as worsening pain (1.6 ± 1.0 vs. 1.4 ± 1.1; *p* < 0.05), weakness (2.1 ± 0.8 vs. 1.4 ± 0.8; *p* < 0.001), loss of appetite (1.5 ± 1.1 vs. 0.8 ± 0.8; *p* < 0.001), tiredness (1.9 ± 0.9 vs. 1.4 ± 0.7; *p* < 0.001), assistance with activities of daily living (1.4 ± 0.98 vs. 0.8 ± 0.7; *p* < 0.001) and organisation of care (0.9 ± 0.9 vs. 0.5 ± 0.6; *p* < 0.01). Other symptoms did not differ regarding time of referral. Additionally through 2013 to 2020 there were no difference in symptom burden (early referral) over the years (F (1,7) = 1.445; *N* = 152; R^2^ = 0.066; *p* = 0.192).

**Table 4:** Hospice and palliative care evaluation (HOPE) based on time of referral according to Wadhwa, D et al.^a^ [21]

*HOPE-SP-CL*^*b*^	*Early referral*^*c*^*,* *(N* = *30)*	*Intermediate referral*^*c*^*,* *(N* = *82)*	*Late referral*^*c*^*,* *(N* = *115)*	*Kruskal–Wallis H*	*p*-value
**Pain**	*n* = 30	*n* = 79	*n* = 104	6.187	< 0.05
Mean ± SD	1.37 ± 1.066	1.22 ± 0.929	1.59 ± 1.03		
**Nausea**	*n* = 29	*n* = 80	*n* = 106	0.756	0.685
Mean ± SD	0.69 ± 0.806	0.65 ± 0.781	0.78 ± 0.894		
**Vomiting**	*n* = 29	*n* = 79	*n* = 104	1.760	0.415
Mean ± SD	0.34 ± 0.553	0.35 ± 0.680	0.51 ± 0.812		
**Dyspnoea**	*n* = 29	*n* = 78	*n* = 106	5.410	0.067
Mean ± SD	1.0 ± 0.964	0.86 ± 0.817	1.16 ± 0.906		
**Constipation**	*n* = 28	*n* = 78	*n* = 102	0.879	0.644
Mean ± SD	0.75 ± 0.844	0.72 ± 0.82	0.63 ± 0.783		
**Weakness**	*n* = 28	*n* = 78	*n* = 105	19.082	< 0.001
Mean ± SD	1.39 ± 0.786	1.64 ± 0.821	2.05 ± 0.825		
**Loss of appetite**	*n* = 29	*n* = 81	*n* = 105	19.705	< 0.001
Mean ± SD	0.76 ± 0.786	0.9 ± 0.903	1.50 ± 1.084		
**Tiredness**	*n* = 26	*n* = 80	*n* = 105	17.662	< 0.001
Mean ± SD	1.35 ± 0.689	1.39 ± 0.803	1.87 ± 0.889		
**Depression**	*n* = 29	*n* = 79	*n* = 98	1.172	0.557
Mean ± SD	1.03 ± 0.865	0.87 ± 0.939	0.96 ± 0.919		
**Anxiety**	*n* = 28	*n* = 81	*n* = 101	0.328	0.849
Mean ± SD	1.07 ± 0.858	0.99 ± 0.942	1.01 ± 0.933		
**Tension**	*n* = 29	*n* = 78	*n* = 98	0.854	0.652
Mean ± SD	0.93 ± 0.842	0.9 ± 0.934	0.8 ± 0.849		
**Wound care**	*n* = 29	*n* = 79	*n* = 99	2.575	0.276
Mean ± SD	0.31 ± 0.660	0.42 ± 0.778	0.24 ± 0.591		
**ADLs**^d^	*n* = 29	*n* = 81	*n* = 101	17.791	< 0.001
Mean ± SD	0.79 ± 0.675	0.93 ± 0.863	1.44 ± 0.984		
**Confusion**	*n* = 28	*n* = 77	*n* = 97	1.738	0.419
Mean ± SD	0.11 ± 0.315	0.08 ± 0.315	0.16 ± 0.472		
**Organisation of care**	*n* = 29	*n* = 75	*n* = 95	9.691	< 0.01
Mean ± SD	0.45 ± 0.572	0.56 ± 0.663	0.89 ± 0.856		
**Overburdening of family**	*n* = 29	*n* = 76	*n* = 90	7.575	0.023
Mean ± SD	1.34 ± 0.857	1.03 ± 0.748	1.33 ± 0.793		
**Global sum score**	*n* = 30	*n* = 81	*n* = 107	12.669	< 0.01
Mean ± SD	13.03 ± 5.404	13.14 ± 5.616	16.08 ± 6.433		
Median (IQR)	11.0 (17.25–9.0)	12.0 (17.0–9.0)	16.0 (20.0–12.0)		

^a^Based on Kruskal–Wallis test. Evaluation from 20 march 2021

^b^HOPE: Hospice and Palliative Care Evaluation Symptom and Problem Checklist

^c^Timing of Referral: early, intermediate and late (> 12, 3–12 and < 3 months before death)

^d^ADLs: assistance with activities of daily living

### Analyses of the laboratory parameters

The laboratory parameters analysed differed significantly when comparing referral groups. As illustrated in Table 5*,* the leukocytes (9.1 ± 5.4 vs. 6.8 ± 4.7; *p* < 0.01), the neutrophil granulocytes (7.4 ± 5.2 vs. 4.8 ± 4.1; *p* < 0.001), the CRP (7.8 ± 7.7 vs. 1.2 ± 1.8; *p* < 0.001), the NLR (10.1 ± 13.9 vs. 4.9 ± 3.4; *p* < 0.001) and the LDH (479.9 ± 508.9 vs. 277.7 ± 182.6; *p* < 0.001) were significant higher in the late referral group. Other objective parameters like haemoglobin (10.3 ± 1.9 vs. 11.8 ± 1.4; *p* < 0.001), total protein (6.1 ± 0.8 vs. 6.6 ± 0.4; *p* < 0.01) and albumin (3.5 ± 0.5 vs. 4.2 ± 0.4; *p* < 0.001) were significantly lower in the group of late referrals.

**Table 5:** Laboratory parameters based on time of referral^a^

*Value*	*Early referral,* *(N* = *30)*	*Intermediate referral,* *(N* = *82)*	*Late referral,* *(N* = *115)*	*Kruskal–Wallis H*	*p*-value
**Leukocytes**^b^	*n* = 29	*n* = 79	*n* = 113	11.932	< 0.01
Mean ± SD	6.834 ± 4.648	6.687 ± 3.547	9.118 ± 5.426		
**Neutrophil granulocytes**^b^	*n* = 29	*n* = 73	*n* = 105	18.068	< 0.001
Mean ± SD	4.767 ± 4.081	4.799 ± 3.304	7.362 ± 5.219		
**NLR**^c^	*n* = 29	*n* = 73	*n* = 105	15.746	< 0.001
Mean ± SD	4.999 ± 3.391	5.662 ± 4.582	10.124 ± 13.877		
**Haemoglobin**^d^	*n* = 29	*n* = 79	*n* = 113	20.854	< 0.001
Mean ± SD	11.817 ± 1.421	10.981 ± 1.692	10.287 ± 1.857		
**Total protein**^d^	*n* = 29	*n* = 78	*n* = 109	11.849	0.003
Mean ± SD	6.582 ± 0.439	6.357 ± 0.720	6.137 ± 0.813		
**Albumin**^d^	*n* = 17	*n* = 46	*n* = 66	23.330	< 0.001
Mean ± SD	4.15 ± 0.364	3.815 ± 0.452	3.533 ± 0.520		
**CRP** [mg/dL]	*n* = 29	*n* = 78	*n* = 112	62.976	< 0.001
Mean ± SD	1.217 ± 1.761	2.976 ± 5.293	7.812 ± 7.741		
**LDH** [U/l]	*n* = 29	*n* = 79	*n* = 112	27.294	< 0.001
Mean ± SD	277.72 ± 182.602	267 ± 100.048	479.94 ± 508.880		

^a^Based on Kruskal–Wallis test. Evaluation from 20 march 2021

^*b*^*Quantity per nanoliter*

^c^*Neutrophil*-to-lymphocyte *ratio*

^d^in g/dL

## Discussion

Although a clear recommendation for early integration of palliative care has already been described, the optimal time point of referral remains unclear [39]. Therefore, we conducted a retrospective analysis to identify factors associated with an “early”, “intermediate” and “late” referral to a SPCC. Our study showed that still most of advanced cancer patients were referred late (< 3 months before death) in the course of their disease. This is in line with the study by Wentlandt et al., who described referral practices of Canadian oncologists to specialized palliative care and defined characteristics associated with these referrals. Hereby they showed that 83.3% of advanced cancer patients were referred less than 6 months before death [40]. Likewise a study by Scibetta et al. [41] showed that from 297 palliative patients 204 (68%) were referred less than 90 days prior to death (late referral). A current study from 2020 by Hausner et al. [13] compared timing of referral before and after the publication of ASCO recommendation supporting early palliative care referral [42]. They showed that late referrals (less than 6 months to death) decreased from 68.8% to 44.8%. However, late referrals were still the majority in both groups. Therefore, further attempts should be made to reach out an early referral that might benefit our patients and their families. In our study, the median time from first time of referral to death of all referrals was 6.38 months, which is better than in other published studies [35, 39]. Since 2013, our oncological CCC has integrated a specialised palliative care consultation (SPCC) for outpatients into their routine care as a measure of quality of care, which might be the reason for this result.

Our analysis clearly showed that pain, social care planning problems and psychological distress were indicators for referring to a SPCC among outpatients with advanced cancer. This data again shows the importance of a proper coverage of palliative care needs, where physical symptoms might not be the main burden of our patients and their families. These results are in line with a systematic review showing that psychological distress is a common recurrent referral criteria for outpatient palliative cancer care (62). Additionally, a low ECOG Performance Status is an indicator for early referral likewise to the study by Carrasco-Zafra et al. [43]. Moreover, the quantity of assistance with activities of daily living changed significantly from early to late referral. Both indicators show once more the impact of loss of autonomy in our patients. To our best knowledge, only two other studies reported on cancer patients’ reason for referral to palliative care [32, 44]. However, these studies only made very rough specifications about the reason for referral, divided into only four aspects: palliative planning, end-of-life care and pain control and/or symptom management.

Moreover, we detected a significant higher intensity of symptoms like “pain”, “weakness”, “tiredness”, and “loss of appetite” in late referrals to specialised palliative care. Social problems like “restriction of daily life “, „overburdening of family” and a higher HOPE global sum score were also frequently associated with late referrals. In a previous study from our CCC, we examined needs and requests of cancer patients in the oncology outpatient clinic for palliative care using a patient reported outcome measurement with MIDOS 2 [30]. Symptoms like “depression”, “anxiety” and “weakness” were indicators for outpatient’s wish for referral to a SPCC [31]. In our study, symptoms like depression or anxiety did not result in an early referral similar to the study of Whadhwa et al. [32]. These results might show the difference between the wish of patient and the reasons for a physician to refer a patient to a specialised palliative care consultation hour. Second, a palliative care nurse rated the questionnaire at first referral to the SPCC. Therefore, further analysis comparing results between self-reported and external assessments would contribute to a better understanding and improvement of patient-centred outcomes. Our reported difference in symptom intensity by early and late referrals are in line with Cheung et al. [45] and Whadwa et al. [32]. The first study analysed 1366 outpatients with advanced cancer. In their study, gastrointestinal, lung and breast cancer were the most common primary cancer sites of patients referred to a palliative care cancer center. The most distressful symptoms were “poor general wellbeing”, “decreased appetite” and “fatigue”, similar to our study. In addition, Whadwa et al. [32] used the Edmonton Symptom Assessment System (ESAS) to compare early (> 12 months before death) with late referrals (< 6 months before death). Patients who were referred late showed a significantly worse overall Symptom score as well as the symptoms “tiredness”, “nausea”, “drowsiness”, “loss of appetite” and “overall wellbeing”, similar to most of our results. Therefore, an increasing intensity of these symptoms could be an indicator for a timely referral to specialised palliative care.

Furthermore, our study showed that early referrals were associated with the female gender independently of the special type of cancer. This is in line with a previous study from the authors Kwon et al. [46]. They compared early referrals (expected survival greater than two years) with late referrals and showed that younger age, female gender, alcoholism and head and neck cancer are indicators for an early referral. Also, a recently study showed that younger age and gynaecological cancer were more likely to receive a PC referral (63). One reason for the association between the female gender and earlier referral to palliative care may be that women attend cancer screenings more regularly than men, as stated by the German health insurance provider “BARMER GEK” [47]. Furthermore, the Robert Koch Institute (RKI), one of the leading biomedical research institutions of the German government, presented similar results years before [48]. Additionally, a further study showed that female gender is more frequently associated with suffering from depression and fear [49–51], so this could be an explanation for the previously mentioned correlation between psychological distress and younger age as indicator for a timely referral.

In contrast, the presence of ascites in cancer patients is an indicator for a late referral. Many studies documented that malignant ascites correlates with a poor overall prognosis and a deterioration in quality of life [52–54]. For example, a retrospective review of 76 patients with malignant ascites by Mackey et al. [55] from 1996, showed that the median survival was 11 weeks from time of diagnosis. Additionally they showed that the presence of low serum albumin and hepatic metastases were significant indicators of poor prognosis.

Some laboratory parameters like NLR, LDH and CRP have been described as indicators of poor prognosis in oncologic patients [56–60]. The NLR is described as a factor related to systemic inflammation, which is associated with cancer growth. Current studies from 2020 and 2021 have suggested that a high NLR is an indicator of lower rates of progression-free and overall survival in various tumour entities such as breast, lung, gastrointestinal and head-and neck cancers [61–65]. For example, Chen et al. [61] analysed changes in NLR among 101 advanced non-small cell lung cancer (NSCLC) patients undergoing therapy with programmed cell death 1 inhibitors. They showed that a high baseline NLR (defined as greater than 4.5) and increased post-treatment NLR were associated with significant increased risk of death and disease progression. In our study we show that Leukocytes, Neutrophils, NLR, LDH and CRP are significantly higher in cancer patients who were referred late to a SPCC. Especially previous studies could proved laboratory data included in prognostic scales are potentially helpful in clinical practice [66]. However, until now they are not integrated in PC routine screening tools, which are frequently used [11, 67, 68]. Furthermore our results show a tendency to use laboratory values as early indicators for first SPCC and not only for the last thirty days before death as currently proven by Stone et al. [69]. Therefore, part of these laboratory parameters should be integrated in palliative care screening tools as measure for identifying appropriate candidates for a specialist palliative care referral.

In sum, our data convincingly supports that patients with late referrals could have received PC earlier, potentially leading to a better outcome. We propose that not only symptom monitoring, but other physical (for example the presence of ascites) and especially laboratory parameters associated with a poor prognosis (such as NLR and low serum albumin) as well, might provide useful information for a timely specialised palliative care consultation. Therefore, its use in the palliative care screening process should be further explored and integrated in actual discussion of one universal screening instrument.

## Limitations

Our study has several limitations. First, the retrospective approach of this study may allow us to miss confounders and generates a sampling bias. Additionally, an adjustment for confounders likewise to the national retrospective cohort study by Allsop et al. [70] is not established, this could leads to different results. Secondly, the results of our monocentric study may not be generalizable. Third, our sample included only patients with oncological diseases; the extent to witch these factors are applicable to patients with non-oncological diseases might be further explored in additional studies. Fourth, the implications of these indicators in the clinical practice and future research should be analysed and discussed in additional studies. Fifth, the characteristics of the referrer were not collected, this might influence early or late referrals given that clinicians who have some palliative care skills or training are more aware of the benefits of palliative care for their patients and families. Sixth, the terms “early” and “late referral” differ between publications, therefore a careful comparison should be made while comparing results from other publications.

## Conclusion

Most patients in an outpatient setting are referred late in the course of their disease. We showed that female gender, having visceral metastasis and psychological distress are mainly reasons and indicators of an early referral. As a new aspect, laboratory parameters and well known prognostic factors like CRP or NLR could be integrated in existing screening tools for oncologist involved in the decision-making about when to refer to a specialized palliative care unit. Further research is required on combining symptoms and laboratory parameters with timely referral to improve the quality of life in advanced cancer.

## Supplementary Information

**Additional file 1. **

## Data Availability

Data is available from the corresponding author on reasonable request.
