# Supplementary material for: Palliative care outpatients in a German comprehensive cancer center—identifying indicators for early and late referral
Source: BMC Palliat Care. 2022 Dec 12;21:221. doi: 10.1186/s12904-022-01114-z (PMC9743520; doi:10.1186/s12904-022-01114-z)
Supplement: Supplementary file 1 — Additional file 1. [file 12904_2022_1114_MOESM1_ESM.pdf]

Dokumentation und Qualitätssicherung in der Spezialisierten ambulanten Palliativversorgung (SAPV) für

palliativmedizinische und hospizliche Einrichtungen und Netzwerke

Unter Nutzung der bundesweiten online-Datenbank der Hospiz- und Palliativ-Erfassung (HOPE)

Die Koordinationsgruppe HOPE und ihre Ansprechpartner

In der Koordinationsgruppe sind die Gesellschaften und Förderer als Kooperationspartner sowie die Berufsgruppen repräsentiert, zusätzlich hat jedes Mitglied inhaltliche Schwerpunkte.

Deutsche Krebsgesellschaft e.V. | Deutsche Gesellschaft für Palliativmedizin e.V. | Deutscher Hospiz- und PalliativVerband e.V.

Prof. Dr. Lukas Radbruch
lradbruch@ukaachen.de

Universitätsklinikum Aachen – Klinik für Palliativmedizin, Medizinische Fakultät

Prof. Dr. Friedemann Nauck
friedemann.nauck@med.uni-goettingen.de

Universitätsmedizin Göttingen UMG – Abteilung Palliativmedizin

Dr. Claudia Bausewein MSc
claudia.bausewein@med.uni-muenchen.de

Dr. Gabriele Lindena
gabriele.lindena@clara-klifo.de

Prof. Dr. Petra Feyer
Petra.Feyer@vivantes.de

Karl Neuwöhner
neuwoehner@hancken.de

Dr. Martin Fuchs
martinfuchs_K@web.de

Dr. Christoph Ostgathe
christoph.ostgathe@uk-koeln.de

Dr. Ute Heinze
ute.heinze@mundipharma.de

Anita Prescher
prescher@krebsgesellschaft.de

Norbert Krumm
nkrumm@ukaachen.de

Josef Roß
josef.ross@pius-hospital.de

Stand: 8.12.2008

1. Einleitung

Die Spezialisierte Ambulante Palliativversorgung (SAPV) will für alle Patienten ein Recht auf Hospiz- und Palliativversorgung und damit ein ambulantes flächendeckendes Versorgungssystem einführen.

In den entsprechenden Gesetzen (GKV-WSG §37b zum 1.4.2007), den Richtlinien des Gemeinsamen Bundesausschusses (GBA vom 20.12.2007) und den gemeinsamen Empfehlungen der Krankenkassen nach §132d (vom 23.6.2008) haben die Dokumentation, die Evaluation und die Qualitätssicherung einen besonders hohen Stellenwert. Auch hat das Ministerium den GBA zu einer jährlichen Berichterstattung, erstmalig zum 31.12.2009 aufgefordert.

Eine solche Berichterstattung ist mit der bisherigen fach-, berufsgruppen- und sektorenbezogenen Dokumentation nicht möglich. Es wird daher eine einheitliche Dokumentation vorgeschlagen, die flächendeckend und gemeinsam in der SAPV genutzt werden soll. Auf dieser Grundlage wird die Vergleichbarkeit aller Initiativen gewährleistet und kann eine Evaluation durchgeführt werden.

Für diese Dokumentation und Evaluation sollte das seit Jahren als Standarddokumentation eingeführte Hospiz- und Palliativerfassungssystem (HOPE) genutzt werden, das für die Anforderungen der SAPV ergänzt wurde.

2. Das Dokumentationssystem HOPE

HOPE wurde 1999 als gemeinsame Dokumentation bei Palliativpatienten mit dem Ziel der Entwicklung einer Standarddokumentation eingeführt. Seitdem wurden in jährlichen Dokumentationsphasen mit wachsender Teilnehmerzahl Erfahrungen ausgetauscht und entsprechende Änderungen am Basisbogen vorgenommen. Parallel zum Basisbogen wurden Module zur vertieften Bearbeitung einzelner Fragestellungen eingesetzt und wesentliche Aspekte daraus in den Basisbogen übernommen.

Der Basisbogen aus HOPE ist eine gemeinsam entwickelte, sektoren- und berufsgruppenübergreifend genutzte, praxisnahe, mit Erfahrungen unterfütterte Standarddokumentation für Hospiz- und Palliativpatienten. In die Entwicklung von HOPE sind Erfahrungen aus dokumentierten Behandlungsverläufen von mehr als 17.000 Patienten eingegangen. In den letzten Erhebungsphasen beteiligten sich zunehmend ambulante Einrichtungen aus allen Bundesländern, in 2008 waren es 45.

Das HOPE-System diente auch als externes Qualitätssicherungsinstrument. Die Teilnehmer haben Qualitätsindikatoren diskutiert, dokumentiert, überprüft. Neben den Berichten an die Teilnehmer wurden die Ergebnisse der Auswertungen in medizinischen Fachzeitschriften veröffentlicht [1-3, 5-8].

Seit 2004 wird in den Erhebungsphasen von HOPE zusätzlich ein Benchmarking zu ausgewählten Indikatoren, z.B. dem Anteil der zu Hause verstorbenen Patienten, durchgeführt.

Die Erfahrungen aus den Auswertungen werden in einem Forschungsprojekt weiter bearbeitet, in dem Qualitätsindikatoren und Dimensionen strukturiert untersucht werden [4]. Die Mitglieder der

Koordinationsgruppe sind an diesem Projekt, das von der Deutschen Krebshilfe gefördert wird, federführend beteiligt. Die Ergebnisse werden in die weitere Entwicklung von HOPE einfließen.

HOPE wird geleitet von einer interdisziplinären multiprofessionellen Koordinationsgruppe aus renommierten Experten zur Dokumentation und Qualitätssicherung in der Palliativversorgung und Vertretern der Kooperationspartner Deutsche Gesellschaft für Palliativmedizin, Deutscher Hospiz- und PalliativVerband und Deutsche Krebsgesellschaft.

Nach den jahrelangen Erfahrungen mit HOPE und der online-Dokumentation ist die Ausgestaltung des Systems als gemeinsame Dokumentations- und Datengrundlage der Hospiz- und Palliativversorgung bundesweit relativ kostengünstig. Die Entwicklung des Dokumentationssystems und der online-Datenbank wird von der Deutschen Krebsgesellschaft, der Deutschen Gesellschaft für Palliativmedizin und dem Deutschen Hospiz- und Palliativverband gefördert und durch eine inhaltlich unbeschränkte finanzielle Zuwendung der Mundipharma GmbH unterstützt.

Mit der Einführung der Spezialisierten Ambulanten Palliativversorgung (SAPV) wurde HOPE erweitert und angepasst. Seit Jahren wurde ein online-Dokumentationssystem angeboten, das nun den beteiligten Einrichtungen ganzjährig zur Verfügung steht. Neu eingeführt wird die Dokumentation für Palliativnetzwerke, bei denen mehrere Einrichtungen gleichzeitig oder nacheinander auf die gleichen Patientendaten zugreifen müssen. Eine Datenspeicherung auf einem gemeinsamen Server und eine automatische Ausgabe der Daten an die Nutzer ist EDV-technisch möglich und sinnvoll.

Die Kooperationspartner empfehlen den Basisbogen als Standarddokumentation für Hospiz- und Palliativpatienten. Für die Evaluation der von der Deutschen Krebshilfe geförderten Palliativprojekte ist 2008 bis 2010/11 neben dem Basisbogen und dem MIDOS-Modul die Dokumentation des Pflegebedarfs mit dem Barthel-Index vorgesehen. Niedersächsische Palliativstützpunkte und das Rheinland haben ihre Dokumentation auf HOPE aufgebaut.

In den ambulanten Hospizdiensten, die überwiegend ehrenamtliche Betreuer koordinieren, bestehen bisher kaum Ansätze zur EDV-Dokumentation. Gemeinsam mit dem Deutschen Hospiz- und PalliativVerband wurde eine Untersuchung mit ambulanten Hospizdiensten durchgeführt, in dem der Koordinator den Basisbogen zur schriftlichen Erfassung des Versorgungsbedarfs des Patienten nutzte. Dies war in den ambulanten Hospizdiensten in der Regel möglich. Damit ist eine Einbindung dieser Dienste in Palliativnetzwerke im Sinne der SAPV auch über die Dokumentation gewährleistet.

Ein Handbuch, das neben dem Konzept Referenzwerte und Zusammenfassungen der veröffentlichten Daten enthält, ist in Vorbereitung.

Im Folgenden werden die Formulierungen aus den gemeinsamen Empfehlungen nach §132 d SGB V aufgegriffen und die Eignung des Dokumentationssystems von HOPE beschrieben.

3. Die Anforderungen an die Dokumentation nach den Empfehlungen des §132 d Absatz 2 SGB V im Einzelnen:

Empfehlungen: 4.4 Die spezialisierten Leistungserbringer haben als Mindestanforderung an die sächliche Ausstattung Folgendes vorzuhalten bzw. sicherzustellen:

i. …für die Spezialisierte Ambulante Palliativversorgung geeignet

Der Basisbogen stellt bei allen Einrichtungsarten und Professionen eine gemeinsame Informationsbasis über die aktuelle Situation von Hospiz- und Palliativpatienten bei Aufnahme, im Verlauf und zu Abschluss der Betreuung dar. Bei jedem Besuch und, wenn sich im Verlauf Änderungen der Situation ergeben oder der Therapie erforderlich sind, sollte eine Spalte im Verlaufsbogen ausgefüllt werden. Zusätzlich sollte der Selbsterfassungsbogen MIDOS zu Beginn und in wöchentlichen Abständen durch den Patienten selbst oder bei einem Interview ausgefüllt werden.

Mit den vorhandenen optionalen Modulen kann HOPE an die Bedürfnisse der ambulanten und stationären Palliativversorgung angepasst werden. Weitere Module für Qualitätssicherung oder für Forschungsfragen können in HOPE integriert und zur Dokumentation und Auswertung für die Dokumentationsphasen allen interessierten Einrichtungen zur Verfügung gestellt werden.

ii. …aktuell geführt

Jede Einrichtung kann online an dem patientenbezogenen Informationsaustausch im jeweiligen Palliativnetzwerk teilnehmen. Die Einrichtungen erhalten dazu jeweils ihren individuellen geschützten Zugang mit Benutzername und Kennwort. Mit dem HOPE-Basisbogen beginnt die patientennahe Dokumentation bei Palliativpatienten. Bei jedem Besuch können mit einer Spalte des Verlaufsbogens aktuelle Informationen eingefügt werden. Der Stützpunkt/ die Koordination kann Telefonprotokolle, Patientenanmeldung und Besuchsstatistik nutzen.

Um nicht nur einen aktuellen Datenabruf, sondern auch eine aktuelle Dateneingabe zu erreichen, können die ambulanten Kooperationspartner mit Table PC oder Handy mit gutem Auflösungsvermögen und Tastatur ausgestattet werden, die eine periphere Dateneingabe ermöglichen.

iii. …für die an der Versorgung Beteiligten jederzeit zugänglich

Jeder frei geschaltete Dienst kann seine Angaben zum Patienten dokumentieren und ggf. ändern. Er kann die Dokumentation anderer in die Behandlung dieses Patienten eingebundenen und frei geschalteten Netzwerkpartner einsehen. Die aktuell eingegebenen Daten werden im gesicherten Datenaustausch online mit der zentralen Datenbank synchronisiert, und so den anderen beteiligten Versorgungseinrichtungen zugänglich gemacht. Die Daten werden zentral auf einem geschützten Server gesammelt. Die Datenübertragung erfolgt mit einer SSL-Datenverschlüsselung wie beim online-banking. Es werden keine Klarnamen verwendet, sondern pseudonymisiert, d.h. alle Dokumente durch eine eindeutige Nummer für jeden Patienten zusammen geführt. Netzwerke/Stützpunkte führen eine Liste mit der Zuordnung der eigenen Patienten zu den verwendeten Nummern. Eine Patientenanmeldung kann nur mit den vollständigen Angaben zu

einem Patienten und nach dessen Einverständnis erfolgen. Ein anderer Versorger erhält Zugriff auf diese Daten mit Leserecht nur, wenn er von dem verantwortlichen Zentrum für diesen Patienten frei geschaltet wurde.

So sollen die notwendigen Informationen unmittelbar nach der jeweiligen Aktion (und der Eingabe) anderen Versorgern zur Verfügung stehen und damit die Koordination der gemeinsamen Versorgung erleichtert werden.

iv. …individuell, mit beteiligten Leistungserbringern abgestimmt

Empfehlungen: 6.4 Die spezialisierten Leistungserbringer erstellen einen individuellen Behandlungsplan, der mit den übrigen an der Versorgung beteiligten Leistungserbringern abzustimmen ist

Die Dokumentation bildet die gemeinsame Informationsgrundlage für alle an einer Patientenbetreuung beteiligten und frei geschalteten Dienste. Alle Einrichtungen nutzen die gleichen patientenbezogenen Formulare und können relevante Informationen bei den anderen Partnern schnell identifizieren. So wird die Therapieplanung und die Koordination der Versorgung erleichtert.

Dabei spielt es zunächst keine Rolle, ob die Netzwerkpartner parallel oder im Anschluss den Patienten betreuen. Bei der Übergabe des Patienten an weiterbehandelnde Netzwerkpartner können jedoch auch ausgewählte Informationen zur Verfügung gestellt werden.

v. …patientenbezogen, sachgerecht und kontinuierlich, als Konzept vorliegend

Empfehlungen: 6.2 Ein geeignetes Dokumentationssystem ist sachgerecht und kontinuierlich zu führen und auf Wunsch der Krankenkasse als Konzept vorzulegen. Das Dokumentationssystem muss patientenbezogene Daten enthalten.

Die HOPE-Datenbank ist mit der Einführung der SAPV ständig offen. Die beteiligten Einrichtungen können damit kontinuierlich dokumentieren.

Das Konzept für die Nutzung von HOPE als Dokumentationssystem in der SAPV kann von der Homepage www-hope-clara.de ausgedruckt und im Sinne der Vorgaben zur Vorlage bei den Krankenkassen genutzt werden.

vi. Qualitätsindikatoren und bundesweite Evaluation

Neben den patientennahen Struktur- und Indikations-, Prozess- und Ergebnisindikatoren sind versorgungsrelevante Aspekte zu prüfen, z.B. welche Strukturen in die Patientenversorgung einbezogen wurden und wie der Informationsaustausch zwischen spezialisierter und nicht-spezialisierter Palliativversorgung funktioniert. Die Erfahrungen und Daten aus HOPE können als Grundlage für die bundesweite Evaluation der SAPV genutzt werden.

vii. …kompatibel mit Dokumentationssystemen

Empfehlungen Absatz 6 Qualitätssicherung: 6.2 Es (das Dokumentationssystem) muss kompatibel zu den bestehenden Dokumentationssystemen der an der Versorgung beteiligten Leistungserbringer sein.

Bei der Vielzahl der Dokumentationssysteme im ambulanten und stationären Sektor ist eine vollständige Einbettung einer Palliativdokumentation kaum möglich. Eine solche Einbettung würde für jedes Dokumentationssystem einen hohen Kostenaufwand bedeuten. Für HOPE wurde ein online-System gewählt, das nicht an Einzellizenzen eines EDV-Programmes gebunden ist und nicht mit lokalen Programmen in Konflikt kommt. HOPE wird zentral aktualisiert, so dass kein Aufwand für lokale Wartung, Aktualisierung oder Fehlerbehebung entstehen kann. HOPE ist inhaltlich ein ergänzendes System zu der üblichen Praxissoftware und Pflegedokumentation.

4. Multiprofessionell vernetzte Versorgungsstruktur

HOPE kann als Plattform für eine gemeinsame Dokumentation die patientenbezogene Kommunikation zwischen den Berufsgruppen ebenso wie zwischen den Netzwerkpartnern unterstützen und die Aktualität der Informationen sichern.

5. Qualitätssicherung

Empfehlungen Punkt 1. Zielsetzung: 1.3 Die Spitzenverbände der Krankenkassen werden die Erfahrungen mit der Umsetzung dieser Empfehlungen fortlaufend auswerten und diese erforderlichenfalls weiterentwickeln.

Empfehlungen Absatz 6. Qualitätssicherung: 6.1 Die spezialisierten Leistungserbringer sind verpflichtet, ein internes Qualitätsmanagement durchzuführen. Sie sollen sich außerdem an Maßnahmen zur externen Qualitätssicherung beteiligen. 6.2 Das Dokumentationssystem muss patientenbezogene Daten und – soweit vorhanden – Indikatoren für eine externe Qualitätssicherung enthalten und eine bundesweite Evaluation ermöglichen.

Das HOPE-System bietet umfangreiche Möglichkeiten zur externen Qualitätssicherung. Die beteiligten Einrichtungen können weiterhin das Benchmarking in den Erhebungsphasen nutzen, in dem zu ausgewählten Indikatoren die eigenen Daten der Einrichtung im Vergleich zur Gruppe von Einrichtungen der gleichen Art dargestellt werden.

6. Optionen

Weitere Optionen können vereinbart werden. So ist zum Beispiel geplant, alle Teilnehmer nach ihrem Einverständnis für eine überregionale Auswertung zur Evaluation nach Einführung der SAPV zu bitten.

7. Datenhoheit und Auswertung

Die Daten aus Netzwerken stehen ausschließlich dem jeweiligen Netzwerk zur Verfügung. Die Einrichtungen können sich ohne zusätzliche Kosten an der jährlichen HOPE-Dokumentationsphase beteiligen. Die Daten aus diesem dreimonatigen Zeitraum gehen dann in anonymisierter Form in einen allgemeinen Datenpool für gemeinsame Auswertungen und als Vergleichswerte für das Benchmarking ein.

8. Literatur

1. Lindena G, Nauck F, Bausewein C, Neuwohner K, Heine O, Schulenberg D, Radbruch L (2005) **Qualitatssicherung in der Palliativmedizin--Ergebnisse der Kerndokumentation 1999-2002.** Z Arztl Fortbild Qualitatssich 99(9): 555-65.

2. Nauck F, Ostgathe C, Klaschik E, Bausewein C, Fuchs M, Lindena G, Neuwohner K, Schulenberg D, Radbruch L (2004) **Drugs in palliative care: results from a representative survey in Germany.** Palliat Med 18(2): 100-7.

3. Nauck F, Radbruch L, Ostgathe C, Fuchs M, Neuwöhner K, Schulenberg D, Lindena G (2002) **Kerndokumentation für Palliativstationen - Strukturqualität und Ergebnisqualität.** Z Palliativmed 3: 41-49.

4. Pastrana T, Junger S, Ostgathe C, Elsner F, Radbruch L (2008) **A matter of definition - key elements identified in a discourse analysis of definitions of palliative care.** Palliat Med 22(3): 222-32.

5. Radbruch L (2005) **Kerndokumentation für Palliativstationen.** Hospiz-Zeitschrift 7: 16-18.

6. Radbruch L, Nauck F, Fuchs M, Neuwohner K, Schulenberg D, Lindena G (2002) **What is palliative care in Germany? Results from a representative survey.** J Pain Symptom Manage 23(6): 471-83.

7. Radbruch L, Nauck F, Ostgathe C, Elsner F, Bausewein C, Fuchs M, Lindena G, Neuwohner K, Schulenberg D (2003) **What are the problems in palliative care? Results from a representative survey.** Support Care Cancer 11: 442-451.

8. Radbruch L, Ostgathe C, Elsner F, Nauck F, Bausewein C, Fuchs M, Lindena G, Neuwohner K, Schulenberg D (2004) **Prozesse und Interventionen auf den deutschen Palliativstationen. Ergebnisse der Kerndokumentation 2001.** Schmerz 18(3): 179-88.

9. HOPE Dokumentationssystem für die SAPV

Funktionen	HOPE SAPV<br>Gesamtpaket für ambulant/stationäre Netzwerke	HOPE Dokumentationsphase 15.3.-15.6.2009	eigene ganzjährige Nutzung
Nutzung und Dokumentation	Angebot	kostenlos während Förderung	kostenlos während Förderung
Koordination Telefonprotokoll	x		
Patientenanmeldung an Kooperationspartner	x		
Patientenbesuchsliste	x		
Basisbogen zur Erfassung des Versorgungsbedarfs, zu Beginn und Ende der Versorgung	x	x	x
MIDOS Selbsterfassung	x	x	x
Verlaufsbogen	x	x	x
Mitarbeiter- und	x	x	x
Angehörigenbefragung	x	x	x
Barthelindex	x	x	
Datenexport	x	x	x
Dateneingabe von Papierdokumentationen	x	mit Abrechnung	
Einrichtung eines Netzwerks zu gemeinsamem Datenzugriff und gemeinsamer Datennutzung und -auswertung (für 20 Nutzer)	x		
Zentrumsverwaltung im koordinierenden Zentrum Stützpunkt)	x		
Automatische Auswertung nach Voreinstellung	x	x	x
Qualitätssicherung	x	x	
Benchmark<br>(für Kriterien wie HOPE, für neue Kriterien)	x	x	
Auswertung und Bericht	x	x	
Vergleich der Regionen	(x)	(x)	
Abrechnungsfunktionen (patientenbezogen nach Besuch, Versorgungsintensität, etc.)	x		
Hotline für Telefonanfragen, inhaltliche, technische Details	x	x	x

10. Anlagen

1. Koordinationsdokumentation
    i. Telefonprotokoll
    ii. Patientenanmeldung
    iii. Besuchsliste
    (bei Verwendung des Verlaufsbogens kann Besuchsliste entfallen)
2. Patientennahe Dokumentation
    i. Basisbogen
    ii. Midos
    iii. Verlaufsbogen
3. Dokumentation im Netzwerk

Telefonprotokoll

lfd. Nr.__
Stützpunkt:__

Telefonat mit:<br>Mehrfachantworten, wenn spezialisiert	Datum<br>online automatisch	Uhrzeit/Dauer	Adresse vom Anfragenden	Thema des Telefonats	Ergebnis/ Maßnahmen	Kürzel
☐ Patient<br>☐ Angehörige<br>☐ Bekannte<br>☐ Pflegedienst<br>☐ Spezialversorger<br>☐ Ehrenamtlicher<br>☐ andere<br>____________<br>____________<br>■ Hospiz<br>☐ ambulant<br>☐ stationär<br>■ Arztpraxis<br>☐ Arzt<br>☐ Arzthelferin<br>■ Krankenhaus<br>Station____<br>☐ Arzt<br>☐ Pflege	|__|__||__|__|20|__|__| Tag Mon. Jahr	Uhrzeit:<br>|__|__|:|__|__| Uhr<br>Stunde Minute<br>Gesprächsdauer:<br>|__|__|__| Minuten	Name: ____________<br>Straße: ____________<br>Ort: ____________<br>Tel..: ____________<br>☐ Erstanruf	☐ Pflegerisch<br>☐ Medizinisch<br>☐ Sozialrechtlich<br>☐ Psychologisch<br>☐ Allgemeine Info<br>☐ Seelsorgerlich<br>☐ andere ____________<br>☐ Beratung<br>____________<br>☐ Info zu Fort- u. Weiterbildung<br>☐ Info zu Veranstaltungen	☐ abgeschlossen<br>☐ vermittelt an:<br>____________<br>☐ Info an:<br>____________<br>☐ Maßnahmen vom Palliativnetz aus<br>____________	
☐ Patient<br>☐ Angehörige<br>☐ Bekannte<br>☐ Pflegedienst<br>☐ Spezialversorger<br>☐ Ehrenamtlicher<br>☐ andere<br>____________<br>____________<br>■ Hospiz<br>☐ ambulant<br>☐ stationär<br>■ Arztpraxis<br>☐ Arzt<br>☐ Arzthelferin<br>■ Krankenhaus<br>Station____<br>☐ Arzt<br>☐ Pflege	|__|__||__|__|20|__|__| Tag Mon. Jahr	Uhrzeit:<br>|__|__|:|__|__| Uhr<br>Stunde Minute<br>Gesprächsdauer:<br>|__|__|__| Minuten	Name: ____________<br>Straße: ____________<br>Ort: ____________<br>Tel..: ____________<br>☐ Erstanruf	☐ Pflegerisch<br>☐ Medizinisch<br>☐ Sozialrechtlich<br>☐ Psychologisch<br>☐ Allgemeine Info<br>☐ Seelsorgerlich<br>☐ andere ____________<br>☐ Beratung<br>____________<br>☐ Info zu Fort- u. Weiterbildung<br>☐ Info zu Veranstaltungen	☐ abgeschlossen<br>☐ vermittelt an:<br>____________<br>☐ Info an:<br>____________<br>☐ Maßnahmen vom Palliativnetz aus<br>____________	
☐ Patient<br>☐ Angehörige<br>☐ Bekannte<br>☐ Pflegedienst<br>☐ Spezialversorger<br>☐ Ehrenamtlicher<br>☐ andere<br>____________<br>____________<br>■ Hospiz<br>☐ ambulant<br>☐ stationär<br>■ Arztpraxis<br>☐ Arzt<br>☐ Arzthelferin<br>■ Krankenhaus<br>Station____<br>☐ Arzt<br>☐ Pflege	|__|__||__|__|20|__|__| Tag Mon. Jahr	Uhrzeit:<br>|__|__|:|__|__| Uhr<br>Stunde Minute<br>Gesprächsdauer:<br>|__|__|__| Minuten	Name: ____________<br>Straße: ____________<br>Ort: ____________<br>Tel..: ____________<br>☐ Erstanruf	☐ Pflegerisch<br>☐ Medizinisch<br>☐ Sozialrechtlich<br>☐ Psychologisch<br>☐ Allgemeine Info<br>☐ Seelsorgerlich<br>☐ andere ____________<br>☐ Beratung<br>____________<br>☐ Info zu Fort- u. Weiterbildung<br>☐ Info zu Veranstaltungen	☐ abgeschlossen<br>☐ vermittelt an:<br>____________<br>☐ Info an:<br>____________<br>☐ Maßnahmen vom Palliativnetz aus<br>____________	

Eingeber Kürzel: A= Arzt, P= Pflege, S= Seelsorge, K= Koordinator, Soz.= Sozialarbeiter E= Ehrenamt

HOPE ®2009 Basisbogen BA Basisbogennr:

Bei Erstkontakt ausfüllen

Patienten – Etikett

2. Geburtsdat.

3. Aufnahme-datum

4. Geschlecht ☐ weiblich ☐ männlich

5. Wohn-situation (nur eine Antwort): ☐ allein ☐ Heim ☐ mit Angehörigen ☐ Sonstige:

Bei Erstkontakt, später nur bei Änderung ausfüllen

6. SAPV

☐ Beratung ☐ additive Teilversorgung
☐ Koordination ☐ vollständige Versorgung

8. Absprache/ Vorlage

☐ Patientenverfügung
☐ Vollmacht
☐ Betreuungsurkunde

9. Pflegestufe

☐ keine ☐ Stufe I
☐ beantragt ☐ Stufe II
☐ Stufe III + ☐ Stufe III

10. Haupt- und weitere Diagnosen / Probleme: wenn keine ICD bitte Text eintragen

Diagnosedatum (ED)

Hauptdiagnose	ICD-10		ICD-10
	ICD-10		ICD-10
	ICD-10		ICD-10

7. Metastasen

☐ Hirn ☐ Lunge ☐ Leber ☐ Knochen ☐ sonstige:

11. Behandlung, Begleitung

☐ Palliativstation ☐ Hausarzt
☐ Hospiz (stationär) ☐ Ambulante Pflege
☐ Krankenhaus (Andere Station) ☐ Palliativarzt (QPA, APD)
☐ Ambulanz ☐ Palliativpflege (AHPP, APD)
☐ sonstige: ☐ Ehrenamtlicher Dienst (AHG, AHD)
☐ Palliativberatung (AHPB)

12. Funktionsstatus (ECOG)

☐ 0 Normale Aktivität
☐ 1 Gehfähig, leichte Arbeit möglich
☐ 2 Nicht arbeitsfähig, kann > 50% der Wachzeit aufstehen
☐ 3 Begrenzte Selbstversorgung, >50% Wachzeit bettlägerig
☐ 4 Pflegebedürftig, permanent bettlägerig

Bei Aufnahme / Abschluss ausfüllen (ev. auch bei Änderung im Verlauf)

13. Datum der Erfassung

14. Probleme					Bemerkungen
Schmerzen	☐ kein	☐ leicht	☐ mittel	☐ stark	
Übelkeit	☐ kein	☐ leicht	☐ mittel	☐ stark	
Erbrechen	☐ kein	☐ leicht	☐ mittel	☐ stark	
Luftnot	☐ kein	☐ leicht	☐ mittel	☐ stark	
Verstopfung	☐ kein	☐ leicht	☐ mittel	☐ stark	
Schwäche	☐ kein	☐ leicht	☐ mittel	☐ stark	
Appetitmangel	☐ kein	☐ leicht	☐ mittel	☐ stark	
Müdigkeit	☐ kein	☐ leicht	☐ mittel	☐ stark	
Pflegeprobleme wegen Wunden / Dekubitus	☐ kein	☐ leicht	☐ mittel	☐ stark	
Hilfebedarf bei Aktivitäten des tägl. Lebens	☐ kein	☐ leicht	☐ mittel	☐ stark	
Depressivität	☐ kein	☐ leicht	☐ mittel	☐ stark	
Angst	☐ kein	☐ leicht	☐ mittel	☐ stark	
Anspannung	☐ kein	☐ leicht	☐ mittel	☐ stark	
Desorientiertheit, Verwirrtheit	☐ kein	☐ leicht	☐ mittel	☐ stark	
Probleme mit Organisation der Versorgung	☐ kein	☐ leicht	☐ mittel	☐ stark	
Überforderung der Familie, des Umfeldes	☐ kein	☐ leicht	☐ mittel	☐ stark	
Sonstige:	☐ kein	☐ leicht	☐ mittel	☐ stark	

15. Was ist der Grund des Kontaktes, was das Behandlungsziel?

HOPE ©2009 Basisbogen ☐☐ ☐☐ ☐☐☐☐ BA Basisbogennr: ______

Bei Aufnahme / Abschluss ausfüllen (ev. Auch bei Änderung im Verlauf)

16. Medikation bis heute		Medikamente
☐ Nichtopioide	☐ Sedativa / Anxiolytika	
☐ Opioide WHO-Stufe 2	☐ Magenschutz	
☐ Opioide WHO-Stufe 3	☐ Laxanzien	
☐ Koanalgetika	☐ Antibiotika	
☐ Kortikosteroide	☐ Diuretika	
☐ Antidepressiva	☐ Kardiaka / Antihyperten.	
☐ Antiemetika	☐ Sonstige:	
☐ Neuroleptika		

17. Maßnahmen / Prozeduren Begleitung (bei Aufnahme bestehend und Abschluss)

Ehrenamtliche	Koordinatoren / Pflege / Arzt		
☐ Unterstützung für Sterbende	☐ Palliativberatung	☐ enterale Ernährung	☐ Chemotherapie
☐ Unterstützung für Angehörige	☐ Koordination	☐ parenterale Ernährung	☐ Strahlentherapie
☐ Sozialanwaltschaftl. Handeln	☐ Psychische Stützung	☐ Medikamente sc	☐ Transfusion
☐ Auseinandersetzung in Glaubens- und Lebensfragen	☐ Sozialrechtliche Beratung	☐ Medikamente iv:	☐ Pleurapunktion
☐ Hilfe bei Organisation letzter Dinge	☐ Beratung in ethischen Fragen.	☐ Medikamente epidural, intrathekal	☐ Aszitespunktion
☐ Unterstützung in akuten Notsituationen	☐ Angehörigen-Anleitung	☐ Anlage / Wechsel ZVK	☐ Versorgung zentraler Zugänge (ZVK, Port…)
☐ Dasein (Erleben von Gemeinsamkeit)	☐ Angehörigenbegleitung	☐ Anlage / Wechsel DK	☐ Versorgung Stomata (Tracheo, Uro, Ileo, Colo…)
☐ Sitzwachen (z.B. im Sterbeprozess)	☐ Trauerbegleitung	☐ Multimodale Schmerztherapie	☐ Darmspülung
☐ Sonstiges:	☐ Case Management	☐ Physiotherapie	☐ Lagerungsbehandlung (path. Fraktur, Spezialbett…)
	☐ Musiktherapie	☐ Psychotherapie	☐ Wundversorgung (Dekubitus, Fisteln, Tumor..)
	☐ Kunsttherapie	☐ Sonstiges:	
	☐ Sonstiges:		

besonderer Aufwand mit:

18. Welches Problem konnte besonders gut gelöst werden?

18a. Welches Problem konnte nicht ausreichend gelöst werden?

19. Wer hat den Bogen ausgefüllt?

☐ Krankenpflege ☐ Arzt ☐ Ehrenamtl. ☐ Psychologe ☐ Sozialarbeiter ☐ Seelsorger ☐ Anderer

Bei Abschluss ausfüllen

20. Datum (Entlassung / Änderung der Betreuung / Tod) ☐☐ ☐☐ ☐☐☐☐

21. Therapieende	22. Verlegung / Weiterbehandlung		23. Sterbeort
☐ Verstorben	☐ Palliativstation	☐ Hausarzt	☐ zuhause
☐ Verlegung, Entlassung	☐ Hospiz (stationär)	☐ Ambulante Pflege	☐ Heim
☐ Ende der Dokuphase	☐ Krankenhaus (Andere St.)	☐ Palliativarzt (QPA, APD)	☐ Hospiz
☐ Sonstiges:	☐ Ambulanz	☐ Palliativpflege (AHPP, APD)	☐ Palliativstation
	☐ sonstige:	☐ Ehrenamtlicher Dienst	☐ Krankenhaus
		☐ Palliativberatung (AHPB)	☐ nicht bekannt

24. Abschließende Bewertung der Zufriedenheit des Teams mit der Betreuung

für die gesamte Behandlung	☐ sehr schlecht	☐ schlecht	☐ mittel	☐ gut	☐ sehr gut
Finalphase (bei verstorbenen Pat.)	☐ sehr schlecht	☐ schlecht	☐ mittel	☐ gut	☐ sehr gut

HOPE ©2008 MIDOS M Basisbogennr:

Sehr geehrte Patientin, sehr geehrter Patient,<br><br>Sie kennen Ihre Situation selber am Besten. Darum bitten wir Sie, diesen Bogen sorgfältig auszufüllen und die Aussagen so anzukreuzen, wie Sie sie im Augenblick bei sich selber wahrnehmen.<br><br>Vielen Dank für Ihre Mitarbeit!	**1. IDNR** ☐☐ ☐☐ ☐☐ ☐☐ ☐☐<br><br>**13. Datum.** ☐☐ ☐☐ ☐☐☐☐

M1. Bitte kreuzen Sie Ihre **durchschnittliche** Schmerzstärke an.

[0] [1] [2] [3] [4] [5] [6] [7] [8] [9] [10]

Kein Schmerz — stärkster vorstellbarer Schmerz

M2. Bitte kreuzen Sie an, wie **stark** heute **Ihre stärksten Schmerzen** waren.

[0] [1] [2] [3] [4] [5] [6] [7] [8] [9] [10]

Kein Schmerz — stärkster vorstellbarer Schmerz

M3. Bitte kreuzen Sie an, wie **stark** heute **Ihre Beschwerden** sind.

**Müdigkeit**	❑ keine	❑ leichte	❑ mittlere	❑ starke Müdigkeit
**Übelkeit**	❑ keine	❑ leichte	❑ mittlere	❑ starke Übelkeit
**Verstopfung**	❑ keine	❑ leichte	❑ mittlere	❑ starke Verstopfung
**Luftnot**	❑ keine	❑ leichte	❑ mittlere	❑ starke Luftnot
**Schwäche**	❑ keine	❑ leichte	❑ mittlere	❑ starke Schwäche
**Angst**	❑ keine	❑ leichte	❑ mittlere	❑ starke Angst
**Andere:**	❑ keine	❑ leichte	❑ mittlere	❑ starke
**Andere:**	❑ keine	❑ leichte	❑ mittlere	❑ starke

M4. Bitte kreuzen Sie an, wie Sie sich heute **fühlen**:

**Befinden**	❑ sehr schlecht	❑ schlecht	❑ mittel	❑ gut	❑ sehr gut

M5. Bemerkungen:

M6. Selbsterfassung **nicht möglich** wegen:

❑ Sprachproblemen ❑ Schwäche ❑ Kognitiven Störungen ❑ Patient lehnt ab ❑ keine Zeit

HOPE ©2008 Verlaufsbogen V **Basisbogennr:**

Datum (Tag Monat Jahr)									
Uhrzeit (Std:Min)/Handzeichen									
Dauer (Std:Min)									
Ort: Klinik, Palliativstation, Hospiz			☐ Klinik ☐ P ☐ Hospiz	☐ Klinik ☐ P ☐ Hospiz	☐ Klinik ☐ P ☐ Hospiz	☐ Klinik ☐ P ☐ Hospiz	☐ Klinik ☐ P ☐ Hospiz	☐ Klinik ☐ P ☐ Hospiz	☐ Klinik ☐ P ☐ Hospiz
zu Hause ZH, Hausarzt, Pflege			☐ ZH ☐ Hausarzt ☐ Pflege	☐ ZH ☐ Hausarzt ☐ Pflege	☐ ZH ☐ Hausarzt ☐ Pflege	☐ ZH ☐ Hausarzt ☐ Pflege	☐ ZH ☐ Hausarzt ☐ Pflege	☐ ZH ☐ Hausarzt ☐ Pflege	☐ ZH ☐ Hausarzt ☐ Pflege
anderes:									
MIDOS	Schmerz								
	Müdigkeit								
	Übelkeit								
	Verstopfung	VRS:<br>0= kein<br>1= leicht<br>2= mittel<br>3= stark							
	Luftnot								
	Schwäche								
	Angst								
	...								
	...								
	Befinden	VRS<br>0= sehr gut<br>1= eher gut<br>2= eher schlecht<br>3= sehr schlecht							
Art der Erhebung			☐ Fremd ☐ Interview ☐ Selbst	☐ Fremd ☐ Interview ☐ Selbst	☐ Fremd ☐ Interview ☐ Selbst	☐ Fremd ☐ Interview ☐ Selbst	☐ Fremd ☐ Interview ☐ Selbst	☐ Fremd ☐ Interview ☐ Selbst	☐ Fremd ☐ Interview ☐ Selbst
**Bemerkungen**<br>**Therapie**									

Eingeber Kürzel: A= Arzt, P= Pflege, S= Seelsorge, K= Koordinator, Soz.= Sozialarbeiter E= Ehrenamt

Dokumentation im Netzwerk: online - Netzwerkfunktionalitäten

Wer	Thema	Optionen	Anmerkungen
Zen-trum	Anmeldung	online unter www.hope-clara.de<br>Auswahl von Test, ganzjähriger Dokumentation, Projekten und Dokumentationsphase, Angabe der eigenen Struktur (Name und Funktion der Einrichtung) und Kontaktdaten und möglichst der Strukturdaten (Personal, Öffnungszeiten, Patientenversorgung)	„Anmeldung abschließen“, dann erhalten Sie eine email an die von Ihnen angegebene Adresse mit Ihren Zugangsinformationen
	Login	Benutzername und Kennwort werden per email an die von Ihnen angegebene Adresse geschickt	wenn der Zugang verwehrt wird, überprüfen Sie bitte die Schreibweise, am sichersten ist die Kopie der Kennung, da häufig 1 und l verwechselt wird.
		Ansicht nach dem Login	
		LOGIN -- HOME -- Download -- Impressum -- LOGOUT<br>HOPE NDS -- Strukturdaten -- Anmeldung -- Download -- User Area	
alle	Navigation	Export	Sie können Ihre Daten jederzeit "auslesen". Wählen Sie die Dokumentationsinstrumente aus und speichern Sie die Tabelle ab. In jeder Zeile steht ein Dokument zu einem Patienten, in jeder Spalte "Feldnamen", die die Fragen im Bogen charakterisieren. Weitere Informationen erhalten Sie unter "Feldinformationen".
		Strukturdaten	Die Strukturdaten haben Sie bei der Anmeldung schon gesehen, hier können sie aktualisiert oder ergänzt werden
		Anmeldung	nicht mehr erforderlich, wenn Sie bereits eingeloggt sind
		User Area	Hier sind Ihr Benutzername und ihre email-Adresse eingetragen, Sie können Ihr Kennwort ändern
	Aufruf von HOPE NDS		HOPE für Niedersachsen mit besonderen Netzwerkfunktionen
Stütz-punkt	Zentrums-verwaltung		
Liste der Zentren	Ein neues Zentrum anlegen		
		Zentren anlegen, durch Stützpunkt bzw. verantwortliches Zentrum	Zentrumsliste mit Adresse, Ansprechpartner und Telefon, email-Adresse, diese erhält Information über Freischaltung und Zugangskennungen
alle Zentren	Zentrums-übersicht Nr…		
Neuen Patienten anlegen / Zugriff erlangen	Besuchsliste	Telefonliste	

Wer	Thema	Optionen	Anmerkungen
Koor-dination	Telefonliste	Telefonformulare erscheinen in der Übersicht als Liste	an erster Stelle das jüngste Telefonat
		Adresseingabe wird nach Datum und Gruppenzuordnung der Anrufenden sortiert	baut nach und nach eine Liste der Ansprechpartner auf, wenn Adressdaten bereits einmal vorhanden, erlaubt die Suchfunktion mit wenigen Angaben den automatischen Eintrag der Adressdaten in die Liste
		nach dem Status der vereinbarten Maßnahmen sortiert	z.B. bei Anfragenvermittlung zum Nachhaken oder in aktiver Liste zur weiteren Beobachtung behalten

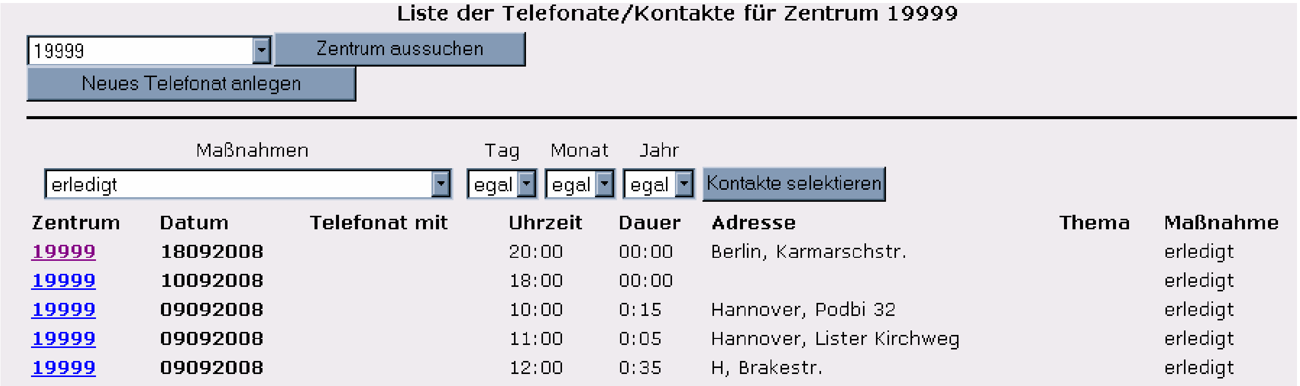

Wer	Thema	Optionen	Anmerkungen
alle	Dokumentation eigener Patienten	Dokumentation starten, „neuen Patienten anlegen“	
		Bei Anmeldung eines Patienten wird geprüft, ob es ähnliche Angaben gibt (Geschlecht und Geburtsdatum), so können Sie einen möglichen Fehler in der Schreibweise finden. Wenn ein Patient mit diesen Angaben schon vorhanden ist, erhalten Sie den entsprechenden Hinweis. Alle anderen Informationen tauchen nicht in der Dokumentation auf (Datenschutz).<br>Den Hinweis auf einen vorhandenen Patienten erhalten Sie nur, wenn alle Ihre Angaben korrekt sind.<br>Bitte notieren Sie sich die vom Rechner vergebene Nummer für die betreffenden Patienten in deren Akte. Dies erleichtert die Suche in der Patientenübersicht.	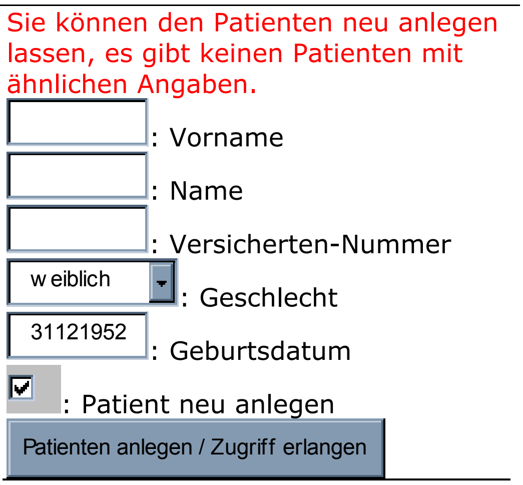 Geburtsdatum und Geschlecht werden geprüft und Hinweis auf ähnliche Patienten gegeben (prüfen, ob bei anderen Angaben verschrieben oder wirklich neuer Patient).<br>Wenn Sie die Zuordnung vergessen haben, können Sie mit allen Angaben für eine Neuanmeldung den Hinweis auf die Patientennummer erhalten.
	Patienten-anmeldung	Vorinformation für Patientenbesuch, Kontaktdaten des Arztes und anderer	Datenauswahl wird in den vor Ort auszufüllenden Basisbogen übernommen

Wer	Thema	Optionen	Anmerkungen
		Dienste erscheinen, Patientenname wird nicht gespeichert!	
	Besuchs-liste zum Eintrag von Patienten-besuchen	Termin, zeitl. Aufwand, davon Pflege und Dokumentation, Ergebnis hier nach Kapiteln, wenn ausführlichere Informationen, dann Wochenbogen oder Basisbogen	als Besuchsliste, dann automatisch Patient zuordnen oder einem Patienten zuordnen und dort eingeben Gesamtdauer automatisch aus Anfahrt zu und Rückkehr vom Patienten (bei Tour beginnt neuer Besuch an der Tür des letzten Patienten)
	Übersichts-zeile je Patient	Bogenart in der Übersichtszeile für Patienten auswählen, in der Zeile darunter erscheinen alle eingegebenen Bögen mit eigenen Farbmarkierungen, rot unterlegt für nicht abgeschlossene Bögen, grün für abgeschlossene Bögen	Dateneingabe mit tab und Zahlentastatur, Häkchen mit Leertaste setzen<br>nach der Dateneingabe oder bei Unterbrechungen der Arbeit immer „speichern“ (Server übernimmt und speichert Ihre Daten), alle Bögen können gezielt nachbearbeitet werden, am Ende des Bogens Häkchen für „Dokumentation abschließen“ anbringen

?	Patient: 2543	18-09-2008 13:43:04	Basis	MIDOS	MA	AN	PB	BA	Abgeschlossen	Archiv	Löschen
?: Versorger suchen: Leisten erscheinen	automatisch vergebene Patnr.	Datum des 1. Aufrufs des Pat	Basis bogen	MIDOS Bogen	Mit-arbeiterbefragung	An-gehörige	Patienten-besuche	Barthel-Index	bei allen ins Archiv	ins eigene Archiv, dort jederzeit aufzurufen	gesamten Patienten löschen, wenn keine Dokumente

Patientendaten ändern	Liste der Zentren mit Zugriffsrechten	Ein Zentrum freischalten	Zwei Patienten zusammenführen

Wer	Thema	Optionen	Anmerkungen
Koor-dination	Liste der Zentren mit Zugriffsrechten	Kontaktdaten aller an der Versorgung beteiligten Zentren sind für alle frei geschalteten Zentren sichtbar	ein neu frei geschaltetes Zentrum erhält per mail eine Information mit Patientennummer des frei geschalteten Patienten, keine Information über Namen und Adresse
		verantwortliches Zentrum kann Patient an Zentrum übergeben, Verantwortung wird abgegeben	
		verantwortliches Zentrum kann Infos für 2 Patienten zusammen führen	alle Daten zu unterschiedlichen Zeitpunkten werden der Patientennummer zugeordnet, von der aus zusammen geführt wird
		Ansicht als frei geschaltetes und verantwortliches Zentrum	nur das verantwortliche Zentrum kann weitere Versorger zuschalten

ZenNr	Name	Ort	Aktion	
19999	testanmeldung	Berlin		
10999	CLARA Test	Kleinmachnow	Zentrum sperren	Patient an Zentrum übergeben

Wer	Thema	Optionen	Anmerkungen
		Zentrum sperren, nur durch verantwortliches Zentrum	Zentrum wird für diesen Patienten abgeschaltet, nur eigene Dokumentation bleibt sichtbar
		Ansicht als frei geschaltetes, aber nicht verantwortliches Zentrum	Bei Rückfragen können Sie die Kontaktdaten des verantwortlichen Zentrums nutzen

ZenNr	Name	Ort	Kontakt	Status
19999	testanmeldung	Berlin	Dr. Test lkjlk lkjlkjlkj - lkjlkj / lkjlk	freigeschaltet
10999	CLARA Test	Kleinmachnow	Dr. Gabriele Lindena - 033203/80858 /	verantwortlich

Wer	Thema	Optionen	Anmerkungen
Koordination	Zentrum für einen bestimmten Patienten ein-beziehen	Zentrumsnummer, Ansprechpartner und Telefonnummer, Funktion des Zentrums erscheinen in der Liste, sowie die Angabe des „verantwortlichen“ Zentrums	alle patientenbezogene Dokumentation wird sichtbar, eigene Dokumentation wird für andere einbezogene Versorger sichtbar<br>Zentrum erhält email mit der Information, dass es in die Versorgung des Pat mit Nr. x einbezogen ist
alle	Dokumentation gemein-samer Patienten	bei frei geschalteten Patientendokumentationen erscheint die eigene Dokumentation zeitlich geordnet zwischen denen der anderen Versorger, alle Dokumente sind mit der jeweiligen Zentrumsnummer gekennzeichnet<br>diese ist in der Liste der aktiven Zentren mit Ansprechpartner und Telefonnummer hinterlegt<br>rot hinterlegte Bögen sind nicht „abgeschlossen“ (Häkchen am Ende jeden Bogens)	die Dokumente anderer Versorger können nicht verändert werden, sie erscheinen im Lesemodus; wenn sich die eigene Beobachtung unterscheidet, muss ein neues Dokument erstellt werden<br>die Bögen können angesehen werden, indem man auf das Datum klickt, ausgedruckt (PDF) und gelöscht werden (Löschen)<br>wenn Sie das Zentrum zur Zentrumsnummer suchen, gehen Sie auf das Fragezeichen in der gelben Patientenzeile
	Patienten-übersicht	alle aktiven Patientendokumente erscheinen in der Patientenübersicht nach Datum geordnet und im Netzwerk mit Zentrumsnummer markiert	Bögen nach Datum sortiert, gekennzeichnet mit Zentrumsnr. des jeweils eingebenden Zentrums (Zuständigkeit für diesen Bogen)

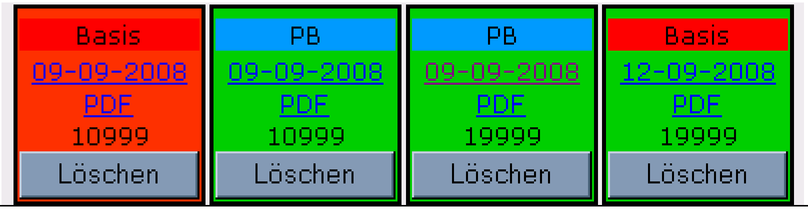

	Änderun-gen am Bogen	nur bei eigenen Bögen	wenn Änderungen der Situation des Patienten beschrieben werden sollen, muss ein neuer eigener Bogen erstellt werden
	Export	Sie können Ihre Daten jederzeit "auslesen". Wählen Sie die Dokumentationsinstrumente aus und speichern Sie die Tabelle ab.	In jeder Zeile steht ein Dokument zu einem Patienten, in jeder Spalte "Feldnamen", die die Fragen im Bogen charakterisieren. Weitere Informationen erhalten Sie unter "Feldinformationen".
	Basisbogen<br>Basis	aktuelle Situation des Patienten, Maßnahmen, Patientenwege, Intensität der Betreuung	neuer Bogen zu Beginn, im Verlauf bei großen Änderungen z.B. des Betreuungsortes, eines Versorgungs-intensitätswechsels und am Ende der Betreuung im eigenen Zentrum
	MIDOS Bogen MIDOS	Selbsterfassung von Symptomen und Befinden durch Patient	bei Schwierigkeiten des Patienten ist ein Interview oder die Befragung von Angehörigen möglich

Wer	Thema	Optionen	Anmerkungen
	Patienten-besuche PB	Termine mit Aufwand und Zeit	Hinweis auf letzten Besuch, wenn kein Basisbogen, dann „gilt“ der letzte aktuelle

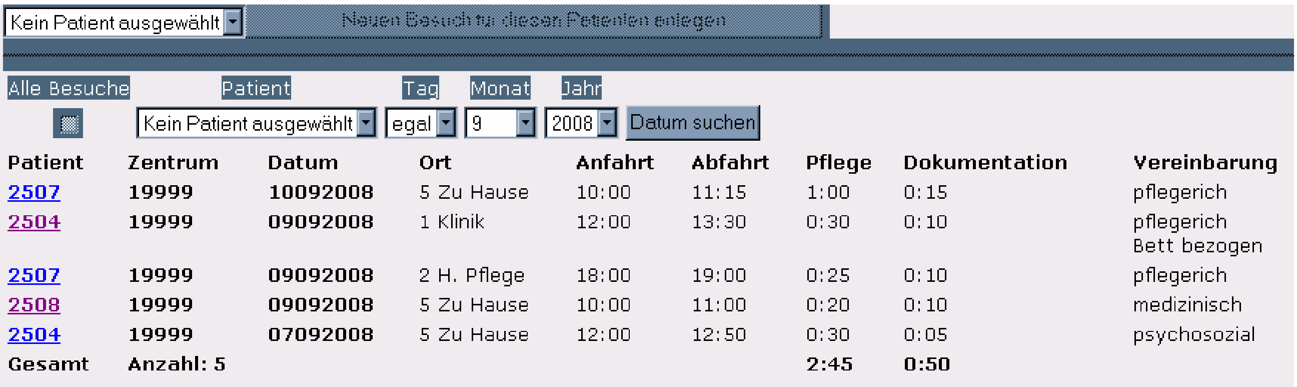

Patient	Zentrum	Datum	Ort	Anfahrt	Abfahrt	Pflege	Dokumentation	Vereinbarung
2507	19999	10092008	5 Zu Hause	10:00	11:15	1:00	0:15	pflegerich
2504	19999	09092008	1 Klinik	12:00	13:30	0:30	0:10	pflegerich Bett bezogen
2507	19999	09092008	2 H. Pflege	18:00	19:00	0:25	0:10	pflegerich
2508	19999	09092008	5 Zu Hause	10:00	11:00	0:20	0:10	medizinisch
2504	19999	07092008	5 Zu Hause	12:00	12:50	0:30	0:05	psychosozial
Gesamt	Anzahl: 5					2:45	0:50	

Wer	Thema	Optionen	Anmerkungen
	abge-schlossen	Funktion für alle Versorger, Patient wird bei allen ins Archiv gelegt	die Unterlagen sind jederzeit im Archiv aufzurufen
	Archiv	archivieren = abgeschlossene eigene Patienten kommen ins eigene Archiv	zentrumsbezogen für abgeschlossene Patienten, die Unterlagen sind jederzeit im Archiv aufzurufen
	löschen	eine Patientendokumentation ist nur zu löschen, wenn es keine Dokumente mehr gibt	es können nur zentrumsbezogen die eigenen Dokumente gelöscht werden
alle	Auswer-tungen, Benchmark ing	Liste von vorausgewählten Auswertungskriterien: aus der Information über die teilnehmenden Einrichtungen werden Gruppen gebildet. Deren Daten erscheinen im Vergleich zu den eigenen Daten in diversen Abbildungen	Die Zählung definierter Kriterien erfolgt automatisch. Wenn keine Angabe in dem betreffenden Feld vorliegt, erfolgt keine Listung.
	Struktur-daten	jedes Zentrum sollte bei Teilnahme an der Dokumentationsphase von HOPE möglichst seine Strukturdaten angeben	Strukturdaten können jederzeit wieder aufgerufen und ergänzt oder aktualisiert werden
	Daten-sicherheit	Der Zugang funktioniert mit Benutzernamen und Kennwort, die Datenübertragung mit SSL-Verschlüsselung. Jedes Zentrum greift auf die eigene Patientendokumentation und die frei geschalteter Patienten zu	Patientenklarnamen erscheinen nicht, Patienten wird im System eine Dokumentationsnummer vergeben.
	HOPE-Dokumen-tations-phase	15.3. bis 15.6.2009	zur Auswertung bei CLARA: Am Ende der Dokumentationsphase werden die vorhandenen Daten ausgelesen. Die Einrichtungen mit mehr als 10 Patientendokumentationen erhalten ihre Daten im Vergleich zu den Gruppen von allen Einrichtungen
	Durchgehe nde ganzjährige Dokumenta tion	Anfangs- oder Enddatum nicht vorgegeben	bis zu 30 Patientendokumentationen am 30.6. werden in die gemeinsame HOPE-Auswertung einbezogen
